# Enhanced Interfacial Properties of Carbon Fiber/Polymerization of Monomers Reactants Method Polyimide Composite by Polyimide Sizing

**DOI:** 10.3390/ma17235962

**Published:** 2024-12-05

**Authors:** Chengyu Huang, Jinsong Sun, Zhiwei Liu, Bo Li, Mingchen Sun, Hansong Liu, Yan Zhao, Peng Zhang, Jianwen Bao

**Affiliations:** 1 School of Materials Science and Engineering, Beihang University, Beijing 100191, China; huangchengyu@buaa.edu.cn (C.H.); lzwbuaa@163.com (Z.L.); smc703@126.com (M.S.); 2 AVIC Manufacturing Technology Institute Composite Technology Center, Beijing 101300, China; sunjsbuaa@163.com (J.S.); liuhansongzhfc@foxmail.com (H.L.); 3 School of Optics and Photonics, Beijing Institute of Technology, Beijing 100811, China; libo@cimm.com.cn

**Keywords:** carbon fiber, polyimide resin, interfacial properties, sizing agent

## Abstract

Carbon fiber (CF)-reinforced polyimide (PI) resin matrix composites have great application potential in areas such as rail transport, medical devices, and aerospace due to their excellent thermal stability, dielectric properties, solvent resistance, and mechanical properties. However, the epoxy sizing agent used for traditional carbon fiber cannot withstand the processing temperature of polyimide resin, of up to 350 °C, resulting in the formation of pores or defects at the interface between the fiber and the resin matrix, leading to the degradation of the overall composite properties. To overcome this problem, in this study, a low-molecular-weight thermosetting polyimide sizing agent was prepared and the processability of the sized carbon fiber was optimized by a thermoplastic polyimide. Compared with the unsized carbon fiber polyimide composites, the interfacial properties of the composites after the polyimide sizing treatment were significantly improved, with the interfacial shear strength (IFSS) increasing from 82.08 MPa to 136.27 MPa, the interlaminar shear strength (ILSS) increasing from 103.7 to 124.9 MPa, and the bending strength increasing from 2262.2 MPa to 2562.1 MPa. The sizing agent acts as a bridge between the carbon fiber and polyimide resin, with anchorage and bonding at the interface between the fiber and resin, which are beneficial for enhancing the interface performance of composites.

## 1. Introduction

In comparison to traditional metallic materials, carbon fiber composites exhibit a number of advantageous properties, including a low density, high specific strength and stiffness, corrosion resistance, fatigue resistance, high-temperature resistance, strong designability, and large-area integral molding [1,2,3,4,5,6]. With the advancement of technology, the performance of carbon fiber composite materials continues to improve. Consequently, since the 1960s, carbon fiber-reinforced resin matrix composites have been employed in aerospace and other cutting-edge technology fields, and continue to be the primary application areas of composite materials, driving the advancement of composite materials technology. The development trend of composites is toward higher strength, higher toughness, better temperature resistance, and more sophisticated automated production processes. Among these areas of development, the ability of resin matrix composites to withstand high temperatures is a significant and promising direction for future research.

Polyimide refers to a class of polymers containing an imide ring in the main chain, which can be divided into thermoplastic polyimide (TPI) and thermoset polyimide (TSPI). From the perspective of high temperature, compared with epoxy resin and bismaleimide resin [7,8], thermoset polyimide, which can be used for a long time above 350 °C, has received more research attention [9,10,11,12,13]. Polymerization of monomers reactants method (PMR-type) polyimide is formed by an aromatic diamine, an esterified aromatic dianhydride, and an end-capping agent through a heat-driven condensation reaction. It has the advantages of the low viscosity of the resin solution, solubility in a low-boiling-point solvent, low porosity, and high-temperature resistance of the composite material. Generally, heat-resistant resins are insoluble and difficult to process, while PMR-type polyimide resins do not have such problems [14,15,16]. PMR-type polyimide composites also have the advantages of low porosity and high-temperature resistance. However, carbon fiber-reinforced PMR-type polyimide composites are often used in high-temperature environments; the difference between the coefficient of the thermal expansion of a carbon fiber and resin matrix under high-temperature conditions is large, resulting in the formation of defects at the interface of the composite material that produce a concentration of thermal stresses, which make the interfacial properties of the composites poor. For this reason, a carbon fiber surface sizing modification is usually used to optimize the interfacial compatibility of the carbon fiber and resin, improve the interfacial properties of the composites, and optimize the molding process [17,18,19].

A sizing agent coating on the surface of a carbon fiber has a very good protective effect [20,21,22], and can isolate moisture and impurities in the air to maintain the surface activity of the carbon fiber. In addition, a sizing agent can make up for the defects on the surface of a carbon fiber, which effectively avoids the loss of mechanical properties caused by friction, fuzz, and breakage between fibers. A sizing agent can improve the infiltration of carbon fibers and their surface activity [23,24,25], which are conducive to the subsequent impregnation of the resin matrix [26,27,28], improving the composite molding process [29,30,31,32]. Most of the commercial carbon fiber has been developed for epoxy resin-based composites, and the sizing agent on the surface is mainly based on epoxy resin [21,33,34]. However, an epoxy resin-based sizing agent has poor thermal stability and is not suitable for the high-temperature-resistant resin molding temperature [35,36]. Hassan et al. [37] prepared a PI sizing agent grafted with carbon nanotubes, which improved the wettability, polarity, and roughness of the carbon fiber, and increased the bending strength and interlayer shear strength of the carbon fiber-reinforced polyether ether ketone (CF/PEEK) composites by 63.2% and 70.5%, respectively, compared to the unsized carbon fiber. Zhu et al. [38] used a methyl pyrrolidone solution of polyetherimide to size carbon fiber. The interfacial shear properties of the carbon fiber-reinforced polyvinyl chloride, polycarbonate, polypropylene, and polyetherimide resin matrix composites were improved after sizing. Yuan et al. [39] synthesized an easy-to-purify semi-aliphatic polyimide sizing agent, and the ILSS and bending strength of the final composites were increased by 23.82% and 7.8%. Chen et al. [40] investigated the effect of a polyetherimide (PEI)/graphene oxide (GO) sizing agent on the interfacial properties of CF/PEEK composites. The results showed that the IFSS of the CF/PEEK composites increased from 43.4 MPa to 63.4 MPa after sizing with 10 wt% PEI + GO sizing agent, which was an increase of 44%.

In this work, for the problem of the weak interfacial adhesion between carbon fiber and a polyimide resin matrix, a polyimide sizing agent was prepared to match the heat resistance of a PMR-type polyimide resin, and the viscosity of the sizing agent was adjusted by adding a thermoplastic polyimide component to provide good adhesion to the carbon fiber and to form a good sizing film, effectively improving the interfacial adhesion between the fiber and resin.

The physical and chemical properties of the sizing agent were analyzed by a thermal gravity analysis (TGA), differential scanning calorimetry (DSC), and Fourier infrared spectroscopy (FTIR); the processing performance of the sizing agent was optimized by viscosity tests and carbon fiber fuzz mass tests. An environmental scanning electron microscope (SEM), atomic force microscope (AFM), dynamic contact angle, interfacial shear strength test, interlaminar shear strength test, flexural properties test, and dynamic thermo-mechanical analysis (DMA) were used to quantitatively investigate the effects of the interfacial properties of the sizing agent-coated carbon fiber composites. This work provides a simple and feasible modification method for the interfacial reinforcement of carbon fiber-reinforced PMR-type polyimide composites, which is of great significance for the optimization of the molding process of polyimide resin matrix composites.

## 2. Materials and Methods

### 2.1. Materials

Carbon fiber CCF800H, 12 K, with an average diameter of 5 μm monofilaments, produced by the dry–wet spinning process, was provided by Weihai Expand Fiber Co., Ltd. (Weihai, China). Phenylacetylene-capped PMR polyimide resin (PMR-Type PI) was provided by AVIC Manufacturing Technology Institute Composite Technology Center (Beijing, China). N,N-dimethylacetamide (DMAc) and ethylene glycol were purchased from Beijing Hyundai Oriental Chemical Company Limited (Beijing, China). Pyromellitic dianhydride (PMDA), 2,3,3′,4′-BiphenyLtetracarboxylic (α-BPDA), 4,4′-(1,3-phenylenebis (oxy))dianiline (APB), and 3-Ethynylaniline (APA) were purchased from Guangdong Wengjiang Chemical Reagent Co., Ltd. (Shaoguan, China). All the reagents used were analytical grade and used directly without further treatment.

### 2.2. Synthesis and Coating of Sizing Agent and Preparation of Composites

**Figure 1:**
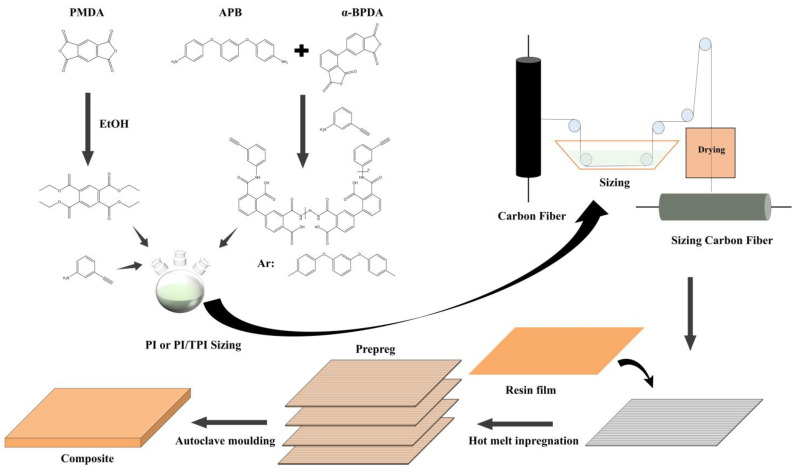
Schematic diagram of synthesis of sizing agents and preparation of polyimide composite materials by sizing carbon fiber.

As shown in Figure 1, PMDA was esterified in ethanol solvent under a nitrogen atmosphere; the system temperature was kept at 80 °C, refluxed under mechanical stirring for 2 h to obtain a transparent and homogeneous organic solution, then APA was added, and a homogeneous and transparent thermosetting amide ester solution was obtained by continued mechanical stirring for 1 h, and the solids content was diluted with ethanol to be 3%, to obtain the thermosetting polyimide sizing agent named PI. To the non-protonic solvent, DMAc, APB, and α-BPDA were added, and the solid content of the system was adjusted to 30%, and the thermoplastic polyimide sizing agent [41], named TPI, was synthesized under conditions of 0 °C, a nitrogen atmosphere, and mechanical stirring. The TPI sizing agent was added to the PI sizing agent, and PI/2% TPI, PI/4% TPI, PI/6% TPI, and PI/8% TPI sizing agents were obtained by adjusting the ratio of the PI to TPI.

The sizing agent prepared above was transferred to a sizing tank for continuous process preparation, and the solvent of the sizing agent was removed by a stepwise temperature increase, with a maximum temperature of 135 °C, to obtain the polyimide sizing carbon fibers, named PI-CF, PI/2%TPI-CF, PI/4%TPI-CF, PI/6%TPI-CF, and PI/8%TPI-CF, respectively. The high-temperature desized carbon fibers were set as the control group, named Desized-CF. Using the hot-melt impregnation method, the carbon fibers were completely impregnated by resin in the prepreg machine through a process of scraping the film, compounding, winding, and cutting, and carbon fiber-reinforced polyimide prepregs with different sizing agents were prepared, controlling for the face density of the carbon fibers to be 133 ± 3 g/m^2^ and the resin content to be 38 ± 3 g/m^2^. The prepregs were laid up according to [0]_16_ (lay 16 layers of unidirectional prepregs in the same direction), and the composite laminates were produced by the hot-press tank molding process (heated to 320 °C, pressurized to 1.5 MPa, and cured at 385 °C for 0.5 h). In addition, epoxy-coated carbon fiber (Epoxy-CF) and desized carbon fiber (Desized-CF) were used to prepare the composites in the same way.

### 2.3. Characterization

#### 2.3.1. General Characterizations

The SEM images were taken by a scanning electron microscope (Quanta 450 FEG, Thermo Scientific Instruments, Waltham, MA, USA) at an accelerating voltage of 10.0 kV. The AFM images were taken by an atomic force microscope (Dimension ICON, Bruker Corporation, Billerica, MA, USA) in tap mode, and the analytical calculations of the surface roughness of the carbon fibers were carried out using software (NanoScope Analysis 3.0). The chemical functional groups of the sizing agents were analyzed by FTIR (Nicolet IS50, Thermo Scientific Instruments, Waltham, MA, USA) in the range of 4000~1000 cm^−1^. The characteristic viscosity of the sizing agents was determined at 25 °C using a capillary viscometer with DMAc as the blank solvent. The fuzz mass of the carbon fibers under different sizing conditions was determined by reference to the Q/AVIC BM 870-2021 standard [42]. The contact angles of the carbon fibers with water and ethylene glycol at room temperature were measured using a dynamic contact angle meter (DCAT 25, DataPhysics Instruments, Filderstadt, Germany). The surface free energy, dispersion component (γ^d^), and polarity component (γ^p^) of the carbon fibers were calculated using the OWRK method.

#### 2.3.2. Interfacial Shear Strength Test

The interfacial shear properties of the carbon fiber monofilament polyimide composites were tested using a Model HM410 Microdroplet Debonding Tester (Beihang University, Beijing, China). The preparation of the microdroplet debonding specimen mainly included hanging the monofilament, hanging the resin microspheres, curing, and other steps as follows: the carbon fiber tow was placed on clean release paper, a monofilament was pulled out randomly and its ends were stuck on the “U” iron sheet, and then the tip of the needle was quickly dabbed with a small amount of polyimide resin solution, and the resin microspheres were dabbed on the monofilament to maintain a uniform size and distribution of the resin microspheres. The size and distribution of the resin microspheres were as uniform as possible. The specimen with resin microspheres was placed in an electric blast-drying oven at 135 °C for 30 min and 380 °C for 10 min, and then cooled naturally after curing.

After the microdebonding specimens were prepared, a microdebonding test was carried out as follows: The U-shaped iron sheet was hung on the instrument, and the resin microspheres with a size of 20–30 μm were selected as the test objects; the instrument was operated so that the blades of the instrument were stuck on the resin microspheres, and the blades were fixed to carry out the debonding test. During the test, the instrument drove the fiber monofilament upwards at a speed of 0.05 mm/min, and the changes in the position of the microspheres were observed through the fiber warp of the instrument, while the force–time curve was recorded by the instrument. The interfacial shear strength (IFSS) was calculated using the following formula:

(1) $$\mathrm{IFSS}=\frac{F_{max}}{\pi dL}$$

where IFSS is the interface shear strength, *F*_max_ is the maximum force at the resin microsphere stripping moment, *d* is the diameter of the carbon fiber monofilament, and *L* is the length of the resin microsphere. Multiple measurements were taken for each sample and at least 8 valid data were obtained.

#### 2.3.3. Composites Interlaminar Shear Strength

The interlaminar shear strength of the carbon fiber-reinforced polyimide composites under the different sizing conditions was tested using a universal mechanical testing machine (INSTRON 5982, Instron Corporation, Canton, MA, USA) with reference to ASTM D2344 [43], with a specimen size of 20 mm × 6 mm × 2 mm, and the ILSS was calculated according to the following equation:

(2) $$\mathrm{ILSS}=\frac{3F}{4bh}$$

where F is the maximum load applied to the sample during the test, *b* is the specimen width, and *h* is the specimen thickness. Multiple measurements were taken for each sample, and at least 6 valid data were obtained.

#### 2.3.4. Flexural Properties Test

The bending strength of the carbon fiber-reinforced polyimide composites under the different sizing conditions was tested using a mechanical universal testing machine (INSTRON 5982, Instron Corporation, Canton, MA, USA) in accordance with ASTM D790 standards. The specimens, with dimensions of 85 mm × 12 mm × 2 mm, were subjected to three-point bending tests. The bending strength (σ_f_) was calculated using the following equation:

(3) $$\sigma_{f}=\frac{3PL}{2bd^{2}}$$

where P is the fracture load of the specimen, *L* is the test span, *b* is the width of the specimen, and *h* is the thickness of the specimen. Each sample was measured several times and at least 6 valid data were obtained.

#### 2.3.5. Thermal Analysis

The thermal stability of the synthetic sizing agents in combination with the polyimide resin was investigated through the use of a thermogravimetric analysis (Discovery TGA5500, TA Instruments, New Castle, DE, USA). A differential scanning calorimeter (Discovery DSC250, TA Instruments, New Castle, DE, USA) was employed to investigate the imidization temperature range of the synthetic sizing agents and polyimide resin. The dynamic thermo-mechanical properties of the carbon fiber-reinforced polyimide composites under the different sizing conditions were evaluated using a Dynamic Thermo-Mechanical Analyzer ((Discovery DMA850, TA Instruments, New Castle, DE, USA), in accordance with standard ASTM D7028, with the specimens measuring 60 mm × 10 mm × 2 mm.

## 3. Results and Discussion

### 3.1. Analysis of PI Sizing Agents

**Figure 2:**
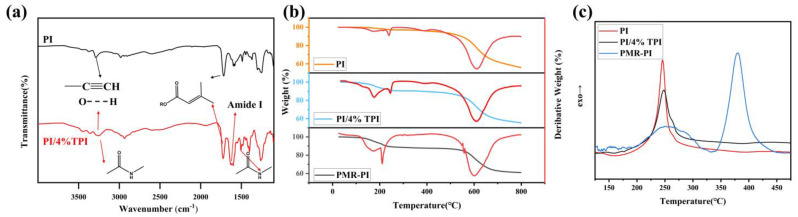
Infrared spectra (**a**), TGA curve (**b**), and DSC curve (**c**) of the sizing agent and PMR-PI resin.

Infrared spectroscopy is a significant method for the qualitative analysis of substances, offering insights into the functional groups of substances and, consequently, their molecular type and structure. The FTIR spectra of the PI sizing agent and the PI/4%TPI sizing agent with the addition of a thermoplastic component are illustrated in Figure 2a, and strong absorption peaks at 1730 cm^−1^ are present in both sizing agents, which correspond with the ester group functionality of the esterification of PMDA (>C=C-COOR). Additionally, there are several weak absorption peaks at 3200~3400 cm^−1^, which correspond with intermolecular and intramolecular hydrogen bonding, as well as with acetylene groups. In comparison to the PI sizing agent, the addition of a thermoplastic component results in the emergence of a new strong absorption peak at 1640 cm^−1^, which can be attributed to the stretching vibration of C=O in the amide I structure. Furthermore, the new absorption peaks at 1506 cm^−1^ and 3250 cm^−1^ are indicative of the bending vibration and the stretching vibration of -NH- in the amide structure, respectively, which derive from the structure of the polyamide acid in the thermoplastic PI sizing agent. These findings indicate that the thermoplastic component TPI sizing agent can be stably present in the thermosetting PI sizing agent system.

After desolvation, the thermogravimetric (TGA) and differential scanning calorimetry (DSC) curves of the two sizing agents, as well as those of the polyimide resin of the PMR type under a nitrogen atmosphere, are presented in Figure 2b and Figure 2c, respectively. From the thermal weight loss data, it can be observed that there is a weight loss interval for both sizing agents and the resin matrix near 200 °C. This corresponds to the stage of the de-esterification of dianhydride and the release of ethanol and water molecules by combining with the amino group in the process of imidization. Furthermore, there is no obvious mass loss after imidization until 550 °C. This indicates that the two sizing agents have good thermal stability. From the DSC curves, it can be observed that the imidization process of the two sizing agents occurs at 200 °C, which matches the imide interval of the resin. This facilitates the diffusion and cross-linking of the sizing agents in the resin during the molding process of the composites. Furthermore, at approximately 380 °C, the active end group (phenylethynyl group) of the resin initiates an addition reaction, forming a three-dimensional cross-linked network structure. This process does not generate by-products, but instead releases a significant amount of reaction heat.

### 3.2. Carbon Fiber Surface Morphology and Infiltration Analysis

**Figure 3:**
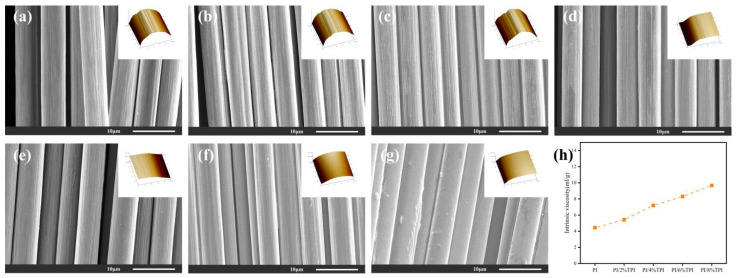
SEM and AFM images of carbon fibers under different sizing conditions: (**a**) Desized-CF; (**b**) Epoxy-CF; (**c**) PI-CF; (**d**) PI/2%TPI-CF; (**e**) PI/4%TPI-CF; (**f**) PI/6%TPI-CF; (**g**) PI/8%TPI-CF; and (**h**) intrinsic viscosity of different sizing agents.

The surface morphology and roughness of the carbon fibers under different sizing conditions were characterized by a scanning electron microscopy (SEM) and atomic force microscopy (AFM), and the fuzz mass of the carbon fibers was tested (Table 1). As shown in Figure 3, the Desized-CF is observed to have multiple and deep grooves with high roughness. The fuzz mass of the fiber is 20.1, which makes the carbon fiber vulnerable to stretching and friction during the winding process, resulting in fiber damage, and ultimately reducing the properties of its composites. This results in a larger porosity of the composite, which in turn affects the mechanical properties of the composite. In contrast, the grooves on the surface of the carbon fiber are repaired by the sizing agent after sizing, which results in a significant reduction in the roughness and the amount of linting of the carbon fiber. The most notable reduction in linting is observed in the PI/4% TPI-CF, with a value of 4.7, accompanied by a notable decline in roughness, from 89.8 to 61.1. In addition, the components and viscosity of the sizing agent (Figure 3h) similarly affect the cohesion, which ultimately influences the efficacy of the sizing agent at forming a surface film on the carbon fiber. When the viscosity is insufficient, the PI/2% TPI is observed to exist in a localized manner on the surface of the carbon fiber, without complete wrapping. Conversely, when the viscosity is excessive, an enrichment phenomenon of the PI/8% TPI is evident on the surface of the carbon fiber, which reduces the opening properties of the carbon fiber. Among the various sizing agents, the PI/4% TPI exhibits the greatest uniformity of spread on the surface of the fiber, thereby forming a stable thin-film layer. This facilitates close contact between the surface of the fiber and the sizing layer, and it serves to minimize abrasion between the fiber and machine rolls, which is beneficial in the prepreg preparation process. Therefore, the PI/4%TPI-CF was taken as the research object for further study.

Good wettability is a prerequisite for the two phases of carbon fiber composites to achieve optimal adhesion. The wettability of a carbon fiber is influenced by two key factors: chemical activity and mechanical engagement. The surface free energy of the carbon fibers was determined through the measurement of the contact angle of the carbon fibers under varying sizing conditions (Table 2). The surface free energy of the desized carbon fiber was found to be the lowest at 31.4 mN/m, indicating that the fiber surface roughness has a limited effect on the gain of fiber infiltration into the resin. The contact angle between the carbon fibers and the tested liquids was significantly reduced after sizing, with the surface free energy of the PI/4%TPI-CF increasing to 47.2 mN/m. This was due to the fact that after the sizing agent was coated, the polar oxygen-containing and nitrogen-containing groups in the sizing layer had good adsorption properties to the liquid, making the carbon fibers more easily infiltrated and permeated by the matrix resin. It is noteworthy that when the thermoplastic component was increased to 6% and 8%, there was essentially no significant increase in the free energy of the fiber surface. This indicates that the surface of the PI/4%TPI-CF had already completely wrapped the sizing agent, and that an increase in the viscosity of the sizing agent did not lead to the better wetting of the fiber by the liquid.

### 3.3. Interfacial Properties of CF/PI Microcomposites

**Figure 4:**
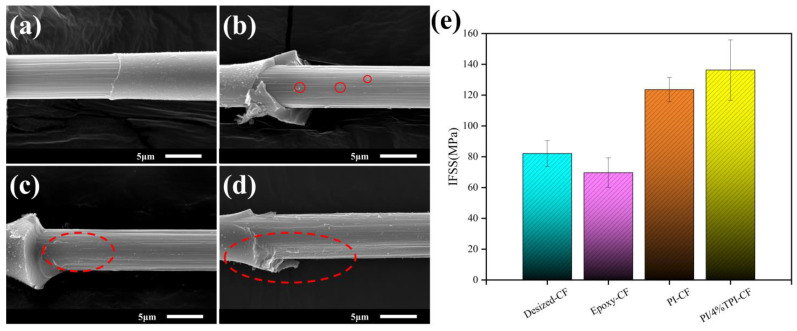
Surface morphology of CFs after PI debonding: (**a**) Desized-CF; (**b**) Epoxy-CF; (**c**) PI-CF; (**d**) PI/4%TPI-CF; and (**e**) IFSS of CF/PI.

A microdebonding test was used to evaluate the interfacial bonding ability between the carbon fibers and polyimide resin matrix under different sizing conditions, and the morphology of the carbon fibers surfaces after the resin microspheres were exfoliated was characterized by SEM. As shown in Figure 4, the grooves on the Desized-CF surface are clear and there is no obvious resin residue (Figure 4a), indicating that only weak intermolecular forces and mechanical embeddedness exist between the resin and the carbon fiber, which lead to the intact and rapid exfoliation of the resin microspheres, and thus present with a small interfacial shear strength, with an IFSS of 82.08 MPa. The Epoxy-CF has poor interfacial bonding with the polyimide resin (Figure 4b), and most of the sizing layer is separated after resin stripping, leaving a separate granular residual resin on the surface of the carbon fiber; this is due to the thermal decomposition of the epoxy sizing agent during the high-temperature curing process of the resin, which leads to the creation of voids between the fiber and the resin matrix, and the bonding between the interfaces during debonding is not sufficient to withstand the stress load of the crack extension, leading to interfacial rapid destruction, resulting in a lower interfacial shear strength (69.62 MPa). In contrast, the micromorphology of the composite interface after sizing with the polyimide sizing agent changes significantly, and more resin fragments remain on the fiber surface of the PI-CF microcomposite after shear damage (Figure 4c), which is conducive to the effective transfer of the load from the resin to the fiber when the interfacial region is subjected to stress, resulting in the IFSS jumping up to 123.59 MPa, an increase of up to 50.6%. The fiber surface of the PI/4%TPI-CF microcomposite has many and continuous resin residues on the fiber surface (Figure 4d), and the irregular jagged shape between the fiber and the resin proves that the interfacial bond between the sizing fiber and the resin is further enhanced, and the IFSS increases to 136.27 MPa, which is 66% higher than that of the control group.

### 3.4. Mechanical Properties of CF/PI Composites

**Figure 5:**
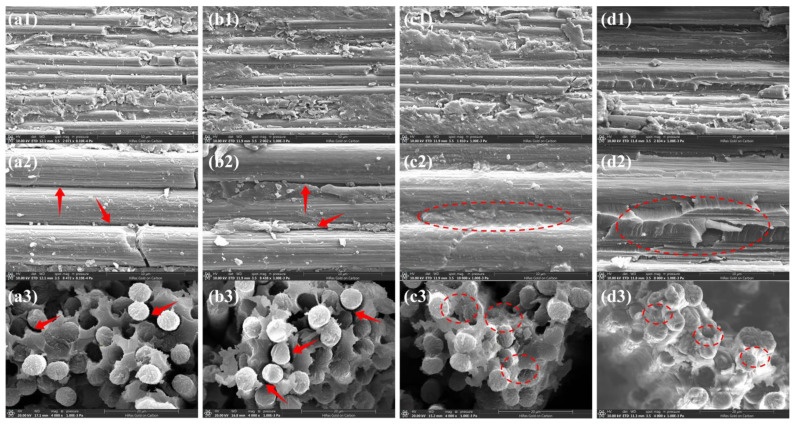
SEM images of fracture of composites after ILSS test and flexural properties test: (**a1**–**a3**) Desized-CF; (**b1**–**b3**) Epoxy-CF; (**c1**–**c3**) PI-CF; and (**d1**–**d3**) PI/4%TPI-CF.

The interlaminar shear strength (ILSS) and bending strength of the composites are also important indexes for evaluating the interfacial properties of the composites, and the SEM images of the failure region taken of the side and cross section of the composites after the test can be used to better study the separation behavior between the carbon fibers and the resin. The Desized-CF/PI composites were found to have a relatively clean surface of the resin (Figure 5(a1–a3)), with no residual resin, which indicates that the interfacial bonding between the fiber and resin was poor, and the interfacial region produced a rigid disruption, with an ILSS and bending strength of 103.7 MPa and 2262.2 MPa, respectively. The Epoxy-CF/PI composites showed more holes and defects in the cross section (Figure 5(b1–b3)), which was due to the poor resistance of the epoxy sizing agents to the high temperatures during the preparation of the composite materials, weakening the mechanical meshing and van der Waals forces between the fiber and resin, resulting in an ILSS and bending strength that were reduced to 90.1 MPa and 2099 mpa, respectively. In contrast, the carbon fiber sized with the polyimide sizing agent was better wrapped by the resin, and the fiber was tightly bonded to the resin (Figure 5(c1–c3)), which prevented debonding between the fiber and the resin, and the resin was able to transfer the load to the fiber well and the failure mode was flexible disruption. In addition, an irregular jagged resin damage morphology existed in the interfacial region of the PI/4%TPI-CF/PI composites, which indicates that the optimized sizing agent could further promote the transfer of stress in the interfacial region, and establish a strong interfacial bond between the carbon fiber and the polyimide resin. The ILSS and bending strength of the PI/4%TPI-CF/PI composites increased to 124.9 MPa and 2562.1 MPa, respectively, with an increase of 20.4% and 13.3%.

**Figure 6:**
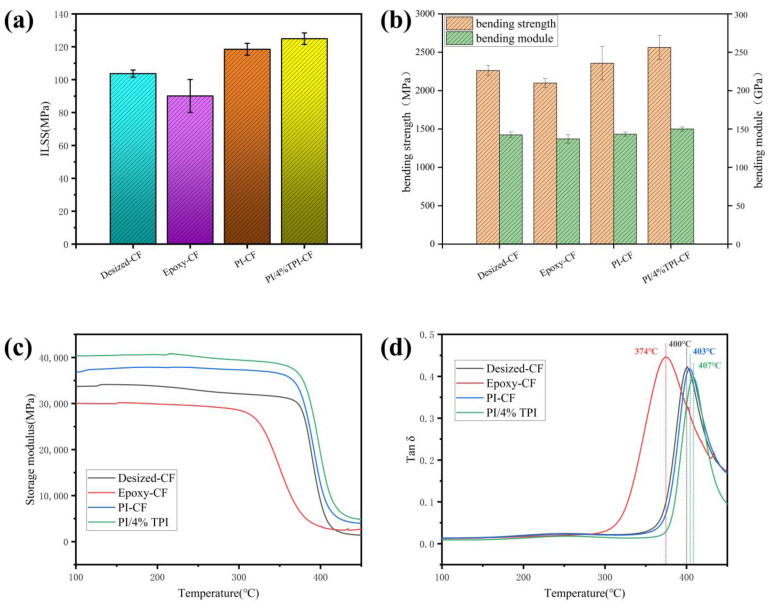
Mechanical properties test of composites under different sizing conditions: (**a**) ILSS; (**b**) bending strength; (**c**) storage modulus in DMA; and (**d**) tan δ in DMA.

The interfacial properties of the composites can also be reflected by the DMA tests, as can be seen in Figure 6c. The energy storage modulus of the Desized-CF/PI composites and Epoxy-CF/PI composites decreases to 34.1 GPa and 30.3 GPa, respectively, whereas the energy storage modulus of the composites is increased to 37.9 GPa and 40.7 GPa after sizing with PI and PI/4% TPI, respectively. From the analysis of the loss factor (Figure 6d), the glass transition temperature (T_g_) of the Desized-CF/PI composites is about 400 °C, while the T_g_ of the Epoxy-CF/PI composite decreases to 374 °C. This is due to the fact that small molecules generated from the decomposition of the epoxy sizing agent are retained inside the composites, which makes the polyimide resin matrix incompletely cured at high temperatures, and the molecular chain is not rigid enough. The PI and PI/4% TPI sizing agents can help the fiber to exert a strong limitation on the resin in the interfacial area; when the temperature reaches the glass transition temperature of the matrix resin, the fiber restricts the chain segment movement of the matrix resin molecular chain at the interface, which is finally reflected in the increase in the glass transition temperature of the composites storage, and the T_g_ increases to 403 °C and 407 °C, respectively.

**Figure 7:**
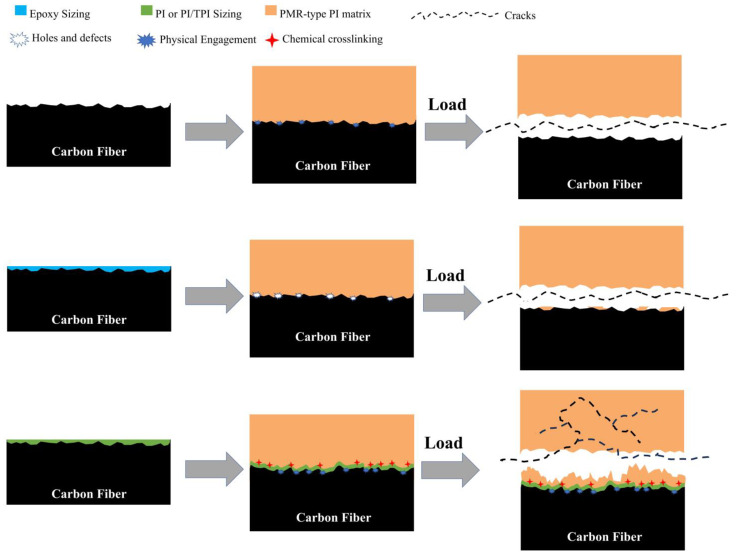
Schematic diagram of interface strengthening mechanism.

Based on the above results, the interfacial reinforcement mechanism of the sizing carbon fiber-reinforced polyimide composites is proposed (Figure 7). For the unsized carbon fiber (Desized-CF), the large number of grooves regularly distributed along the axial direction on its surface are conducive to the interpenetration of the carbon fiber and matrix resin, but the strength of this mechanical engagement is limited, which means that when the composite material experiences stress damage, brittle damage usually occurs at the interface, and the resin and the fiber are completely detached, and the interfacial strength at this time depends only on the anchorage between the fiber and the resin. For the epoxy sizing carbon fiber (Epoxy-CF), because it cannot match the curing process of the high-temperature-resistant polyimide resin, the impurities generated by thermal decomposition during the molding process of the composite material increase the pores and defects between the resin and the fiber, and limit the filling of the fiber during the curing process of the resin, making the failure mode of the composite materials under a load interface detachment. And the adhesion of the fiber to the resin matrix is very small, which shows poor interfacial properties. Polyimide type sizing agent plays the role of connecting carbon fiber and polyimide resin, and can participate in the imidization reaction of resin, which is conducive to the physical diffusion effect and chemical bonding effect of sizing agent on resin during the interface construction of composite materials, which makes the interface adhesion of composite materials prepared by carbon fiber coated with such sizing agent stronger.. In this case, the cracking of the matrix also becomes a part of the interface failure. When the material is loaded, the crack can be transformed into many micro-cracks across a large area, instead of directly generating interfacial debonding. The cracks have to dissipate more energy in the process of dispersion and expansion in the interfacial area to destroy the chemical bond formed between the sizing agent and resin matrix, which ultimately feeds back to the enhancement of the interfacial properties of the composite material.

## 4. Conclusions

In this paper, a polyimide sizing agent that matched the compatibility and heat resistance of a PMR-type polyimide resin was successfully synthesized, and enhanced the coating property of the sizing agent on carbon fibers with the addition of a thermoplastic polyimide component. An amount of 4% of a thermoplastic polyimide was added to the sizing agent to form a homogeneous and continuous coating film on the surface of a carbon fiber, which achieved the coordination of the bundling and opening properties of the carbon fiber. After sizing, the wettability of the matrix resin to the carbon fiber was improved. During the molding process of composite materials, the sizing agent can participate in the imidization of the resin to achieve physical diffusion and chemical cross-linking of the resin matrix, thereby enhancing the interfacial properties of polyimide composite materials. Impressively, the composites’ IFSS, ILSS, and bending strength were increased to 136.27 MPa, 124.9 MPa, and 254.9 MPa after the sizing, respectively, which were 66%, 20.4%, and 13.3% greater, respectively, compared to the desized carbon fiber. An analysis of the damage morphology of the composite interfacial region from the interlaminar shear strength test and bending strength test revealed that the intrinsic factor behind the enhanced interfacial properties of the composite was that the sizing agent could help transfer the load from the resin to the fiber efficiently, and the strong bonding effect between the sizing agent and resin could disperse the cracks, which greatly dissipated the energy required for crack propagation. In addition, thermo-mechanical tests also verified the influence of the sizing agent on the interfacial properties, with a small increase in the glass transition temperature of the carbon fiber-reinforced polyimide resin matrix composites after sizing. This work provides a facile synthesis of polyimide sizing agents in a process that uses low-boiling-point solvents, which can be carried out under mild conditions and can be applied to the large-scale continuous production of polyimide composites.

## Figures and Tables

**Table 1:** Surface roughness and fuzz mass of carbon fibers under different sizing conditions.

Carbon Fiber	Roughness (Ra/nm)	Fuzz Mass (mg)
Desized-CF	89.8 ± 15.8	40.1
Epoxy-CF	75.9 ± 15.6	6.8
PI-CF	84.2 ± 8.3	11.3
PI/2%TPI-CF	72.1 ± 23.8	7.4
PI/4%TPI-CF	61.1 ± 15.2	2.7
PI/6%TPI-CF	57.9 ± 15.4	5.6
PI/8%TPI-CF	50.6 ± 10.3	19.5

**Table 2:** Contact angles and surface free energy of carbon fibers under different sizing conditions.

Carbon Fiber	Contact Angle (°)		γ (mN/m)	γ^d^ (mN/m)	γ^p^ (mN/m)
	Deionized	Ethylene Glycol			
Desized-CF	89.8 ± 15.8	40.1	31.4	16.6	14.8
Epoxy-CF	75.9 ± 15.6	6.8	47.0	10.4	36.5
PI-CF	84.2 ± 8.3	11.3	42.5	14.1	28.5
PI/2%TPI-CF	72.1 ± 23.8	7.4	43.7	11.4	32.3
PI/4%TPI-CF	61.1 ± 15.2	2.7	47.2	7.9	39.3
PI/6%TPI-CF	57.9 ± 15.4	5.6	48.2	10.1	38.1
PI/8%TPI-CF	50.6 ± 10.3	19.5	49.0	9.9	39.0

## Data Availability

The data presented in this study are available upon request from the corresponding author. The data are not publicly available due to the fact that they are also part of an ongoing study.
